# Identifying protein conformational states in the Protein Data Bank: Toward unlocking the potential of integrative dynamics studies

**DOI:** 10.1063/4.0000251

**Published:** 2024-05-17

**Authors:** Joseph I. J. Ellaway, Stephen Anyango, Sreenath Nair, Hossam A. Zaki, Nurul Nadzirin, Harold R. Powell, Aleksandras Gutmanas, Mihaly Varadi, Sameer Velankar

**Affiliations:** 1 Protein Data Bank in Europe, European Bioinformatics Institute, Hinxton, United Kingdom; 2 The Warren Alpert Medical School of Brown University, Providence, Rhode Island 02903, USA; 3 Imperial College London, Department of Life Sciences, London, United Kingdom; 4 WaveBreak Therapeutics Ltd., Clarendon House, Clarendon Road, Cambridge, United Kingdom

## Abstract

Studying protein dynamics and conformational heterogeneity is crucial for understanding biomolecular systems and treating disease. Despite the deposition of over 215 000 macromolecular structures in the Protein Data Bank and the advent of AI-based structure prediction tools such as AlphaFold2, RoseTTAFold, and ESMFold, static representations are typically produced, which fail to fully capture macromolecular motion. Here, we discuss the importance of integrating experimental structures with computational clustering to explore the conformational landscapes that manifest protein function. We describe the method developed by the Protein Data Bank in Europe – Knowledge Base to identify distinct conformational states, demonstrate the resource's primary use cases, through examples, and discuss the need for further efforts to annotate protein conformations with functional information. Such initiatives will be crucial in unlocking the potential of protein dynamics data, expediting drug discovery research, and deepening our understanding of macromolecular mechanisms.

## INTRODUCTION

As of February 2024, the Protein Data Bank (PDB),^1^ the global repository of experimentally determined structures, hosts over 215 000 macromolecular structures. Recent advances in protein structure prediction—made by the new generation of AI-based tools such as AlphaFold2,^2^ RoseTTAFold,^3^ and ESMFold^4^—have predicted almost 1 × 10^9^ further structures, archived in the AlphaFold Protein Structure Database (AFDB),^5^ the ESM Metagenomic Atlas,^4^ and the Model Archive.^6^ Although significant work is ongoing to generate ensemble models, these tools generally predict a single structure per sequence.^7^

**FIG. 1.**
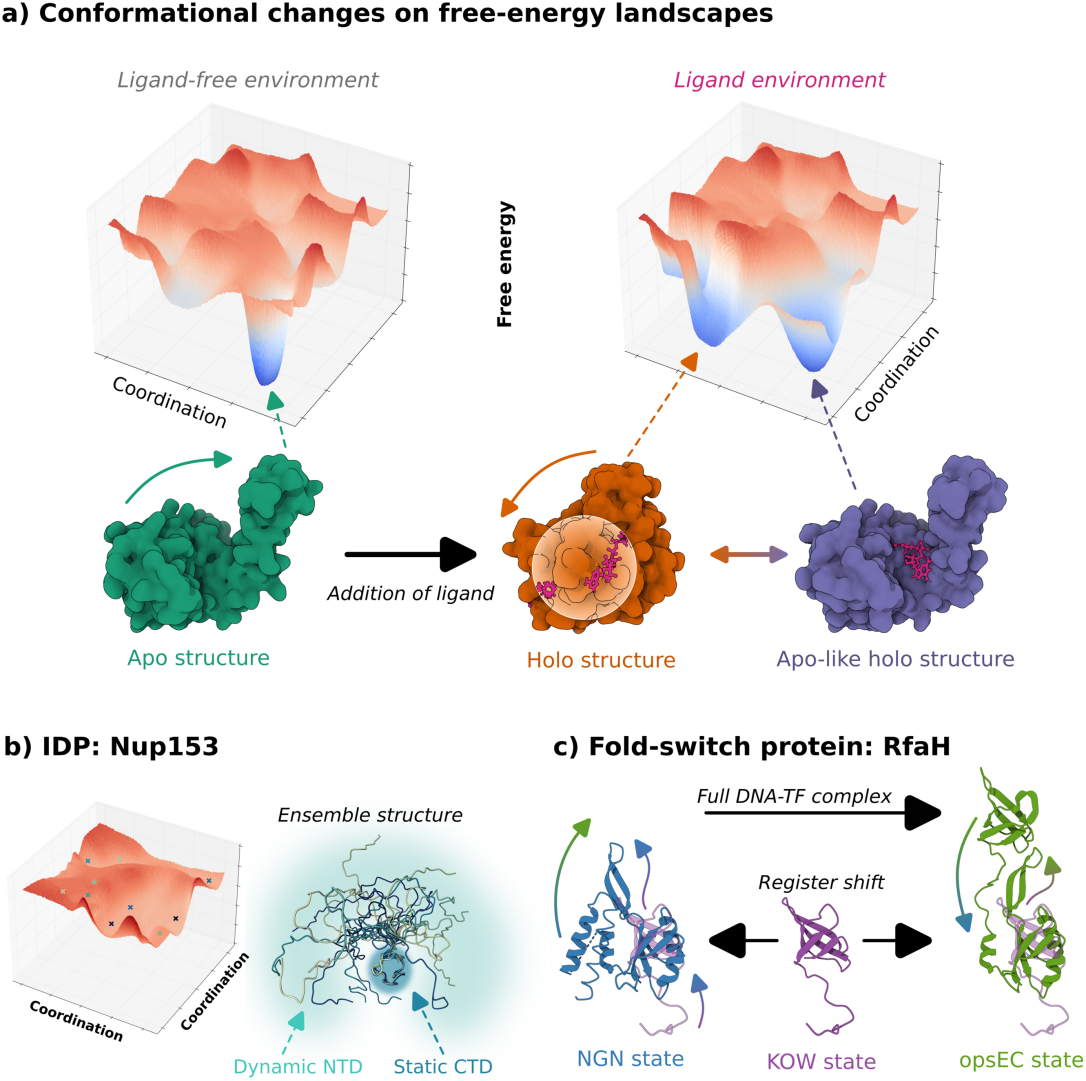
Illustration of functional protein conformation changes. (a) Hypothetical free-energy landscape (top) of adenylate kinase's coordination state before (left) and after (right) ligand binding. A dominant minimum is plotted in the ligand-free environment, facilitating one apo conformation (PDB: 6S36, green). Addition of ADP changes the landscape to accommodate a second minimum for the adoption of a closed conformation (PDB: 8CRG, orange), while permitting the existence of the original conformation (PDB: 6F7U, indigo). An energy barrier between these states must be overcome to transition between the conformations. (b) Hypothetical free energy landscape of the human nuclear pore complex protein Nup153—an IDP (PDB: 2EBV). (c) Conformational states of *E. coli* transcription factor RfaH binding the NusG N-terminal domain (NGN, left), the NusG C-terminal domain (KOW, middle), and when bound to an operon polarity suppressor (*ops*) DNA sequence in the transcription elongation complex (opsEC, right).^33^ KOW-bound structure is truncated—solved for the N-terminal domain only. PDB: 2OUG, blue; PDB: 2LCL, purple; PDB: 6C6S, green.

To realize the relationship between protein sequence, structure, and function, we must consider their dynamics—relative movements between residues. The structure of a protein navigates a high-dimensional conformational landscape, where stable conformations occupy free energy minima.^8^ The transitions between these minima represent conformation changes, often crucial for protein function, both under physiological conditions or in disease progression.^9,10^ Changes to the landscape's topology may be induced via ligand association, solvent packing, oligomerization, pH changes, or post-translational modification^11–16^ [Fig. 1(a), right]. On the far end of the conformational flexibility spectrum are the intrinsically disordered proteins (IDPs), whose free energy landscapes lack deep energy minima [Fig. 1(b)], instead being littered with shallow dips that could become more favorable upon environment changes.^17–19^ Investigating these landscapes requires diverse experimental techniques, each contributing unique insights into conformational states or motion of proteins^20–22^ [Fig. 1(c)].

X-ray crystallography has been instrumental in providing atomic-resolution models of proteins. Despite its tendency to capture proteins in static states due to crystal packing, advancements such as temperature-jump and time-resolved serial femtosecond crystallography (SFX) can observe local dynamic processes within crystallized proteins.^23–27^ In contrast, small-angle x-ray scattering (SAXS) is a low-resolution method for studying larger, global conformation changes in solution.^28–31^ Combined with experimentally derived or predicted atomic models, integrative SAXS models can offer impressively comprehensive views of macromolecular states and dynamics.^24,32^

In recent years, cryogenic electron microscopy (cryoEM) has dramatically improved to solve thousands of macromolecules—particularly those that are difficult to crystallize.^34^ Like x-ray crystallography, cryoEM has traditionally produced single, static models from three-dimensional (3D) projections of many images.^35,36^ However, advances in direct-electron detectors and image classification software facilitate the reconstruction of conformational ensembles,^37–41^ offering views of proteins in states closer to their physiological conditions. Furthermore, the study of individual molecules from cryo-electron tomography (cryoET) reveals the conformation of macromolecules *in situ.*^42–47^ Such physiological insight was once the preserve of nuclear magnetic resonance (NMR) spectroscopy, which excels at detailing structure and dynamics over a range of timescales in near-physiological conditions.^48–51^ NMR can detect both transient states^52^ and intrinsically disordered regions,^53,54^ providing insight into protein movements and interactions crucial for biological function. However, its application is typically limited to smaller proteins and complexes—complementing the data collected by cryoEM, which struggles to resolve smaller macromolecules.^38,48^

The success of AI-based tools at predicting protein structures from amino acid sequences has marked a significant milestone in structural biology.^2–4^ However, modeling the conformational states of proteins remains a frontier,^55–59^ as demonstrated by the general tendency of AlphaFold2 to predict structures in similar conformations.^56,60–65^ Innovations have emerged where modifications to the multiple sequence alignments (MSAs), a key input for many structure prediction tools, enable the exploration of more diverse protein conformations.^7,61,64,66,67^ For example, the AF-Cluster technique has demonstrated through experimental validation that AlphaFold can predict multiple states of the fold-switching protein KaiB.^65,68^

While structure prediction tools can help investigate conformational heterogeneity, molecular dynamics (MD) simulations remain indispensable for probing the theoretical dynamic behavior of macromolecules, complementing the generally static models provided by AI-based predictions and experimental data.^69–71^ Despite their computational cost and the challenges associated with force field accuracy, MD simulations are invaluable tools for exploring the conformation space and potential biological activities of proteins, helping to identify novel ligand-binding sites crucial for drug discovery.^63,72–74^

Here, we describe the method the Protein Data Bank in Europe – Knowledge Base^75^ (PDBe-KB) uses to aggregate and cluster protein conformational states, primarily from x-ray, cryoEM, and NMR structures deposited in the PDB.

## METHODS

**FIG. 2.**
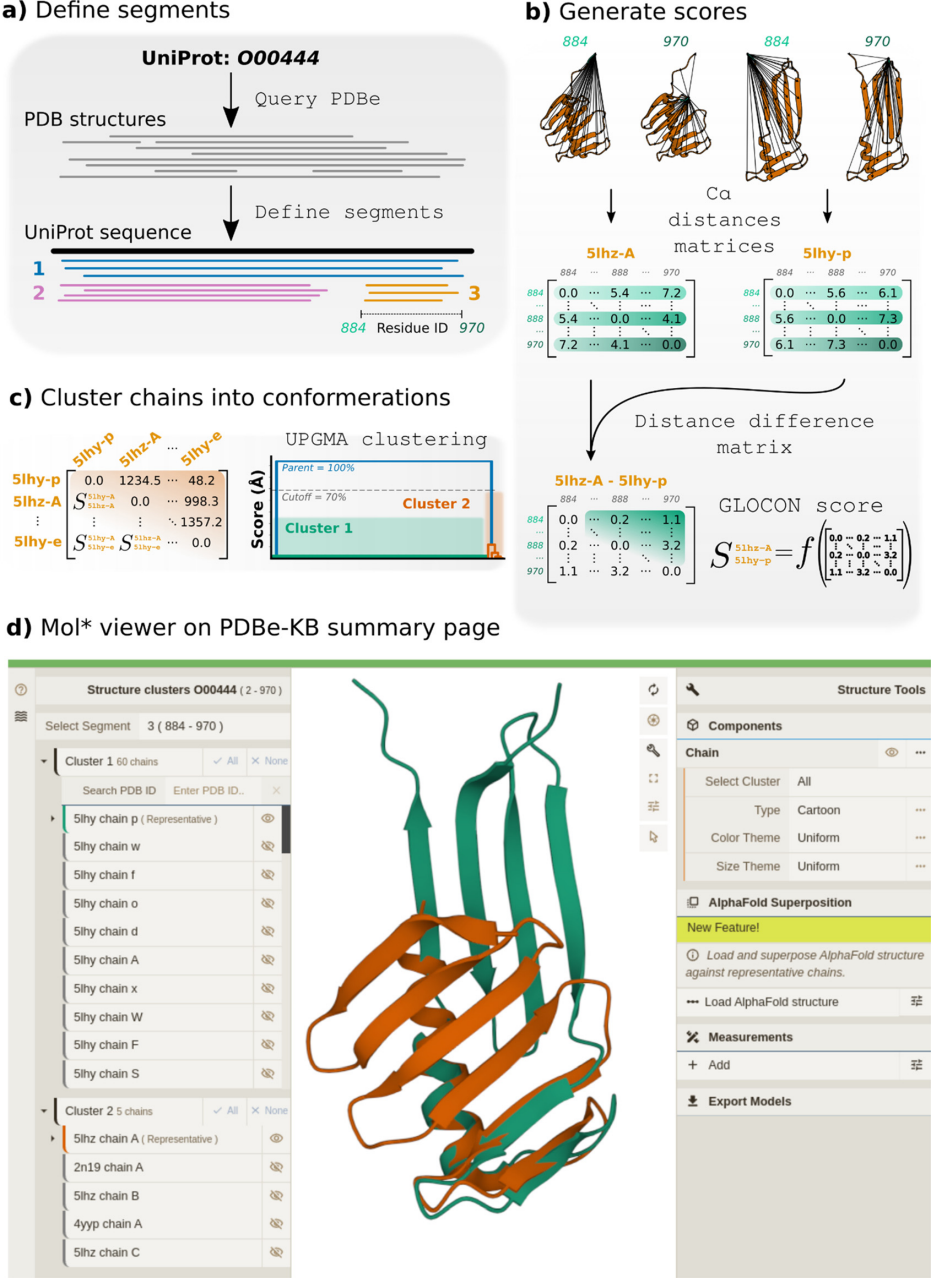
Automated identification of protein conformational states across the PDB archive. **(**a) All chains of a given UniProt accession (100% sequence identity) are assigned to segments based on their overlap with the reference UniProt sequence. Non-overlapping sequences are grouped into separate segments. (b) Chains are superposed to all other chains within their assigned segment. (c) Chain–chain GLOCON scores are calculated for all polypeptides within a segment (refer to Ref. 89 for formal definition) before (d) agglomerative clustering is performed. The results are displayed in 3D on PDBe-KB aggregated views of proteins pages.

The first step of the clustering process is to collate polypeptide chains from the PDB with 100% sequence identity into groups called *segments* [Fig. 2(a)]. A single segment will contain only structures mapping to a contiguous section of their corresponding UniProt sequence, potentially resulting in multiple segments per UniProt sequence (such as truncated N- or C-terminal domains). Each polypeptide in the PDB archive is mapped to a corresponding UniProt sequence using the SIFTS annotation tool.^76,77^ Only chains within segments are subsequently considered for clustering.

Next, we calculate the Euclidean distances between Cα atoms per residue pair, leading to a transformation-independent Cα distance matrix. Polypeptides are compared pairwise by calculating the absolute difference between their Cα distance matrices, capturing the chain–chain differences in Cα position, independent of the chains' original Cartesian coordinates. The distance matrix is filtered by reducing elements to zero if below 3 Å, removing small discrepancies in Cα placement between structures. To condense this filtered difference matrix, the upper diagonal elements are summated and normalized by multiplication with the fraction of modeled residues, penalizing any gaps in the structures [Fig. 2(b)]. This measure captures the GLObal CONformation (*GLOCON*) difference as a dissimilarity score between chains.

Next, we use UPGMA agglomerative clustering to group chains based on their GLOCON scores, splitting the segment into *clusters*—approximating potential conformational states [Figs. 2(c) and 2(d)]. Based on the GLOCON dissimilarity score, small structural differences (such as changes in loop position) are noticeable by this clustering method, such as in the manganese ABC transporter's Leu127-Lys135 region (UniProt accession: P0A4G2). However, small differences could be obscured where small and large differences occur (such as domain movements or fold switches). Reasonable separation into clusters is generally achievable at 70% of the maximum GLOCON score, although this threshold could be further optimized per segment. All chains are superposed (independently of the clustering step) using GESAMT, which identifies structurally conserved regions between possibly heterogeneous structures.^78^ Where NMR structures are clustered, the first model of the ensemble is selected as a reference. PDBe runs this pipeline weekly,^75^ predicting conformations for the entire PDB archive.

Alongside the experimentally derived structures, our process allows users to superpose the corresponding AlphaFold2 model, supplementing the cluster results. The root-mean-squared deviation of the AlphaFold2 model from each cluster's representative chain is calculated and displayed, allowing identification of the conformational state predicted by AlphaFold2. This comparison allows users to quickly identify the conformational state predicted by the full-length AlphaFold2 protein, potentially expediting functional characterization.

To test the clustering pipeline, we manually curated a benchmark dataset of polypeptide chains in the PDB archive that adopt open or closed conformations,^89^ similar to previous datasets characterizing distinct secondary structure changes during fold switching.^79^ An initial search identified 630 unique entries with descriptions of open or closed in their PDB entry title before filtering the results for spurious substrings (e.g., cycl*open*tadienyl). Publications for the remaining 315 entries were read to designate labels of conformational states. The dataset comprises a range of structural variations at different scales, such as a ∼5 Å loop movement in α-fucosidase (UniProt accession: J9UN47), a set of intra-domain rearrangement of residues in NMR structures (e.g., PDB code: 6qeb) of human carbonic anhydrase (UniProt accession: P00918), and a ∼20 Å C-terminal domain movement at 5′-deoxynucleotidase's Glu332 hinge (UniProt accession: P21589). We make the dataset available through the PDBe-KB's FTP server and Kaggle.

All the data from the clustering process are openly accessible from the PDBe-KB FTP area, through API end points in the PDBe Aggregated API and via the PDBe-KB aggregated views of proteins. The code is open source and available on GitHub under the Apache 2.0 license.

## RESULTS: NOTABLE EXAMPLES FROM THE ARCHIVE

**FIG. 3.**
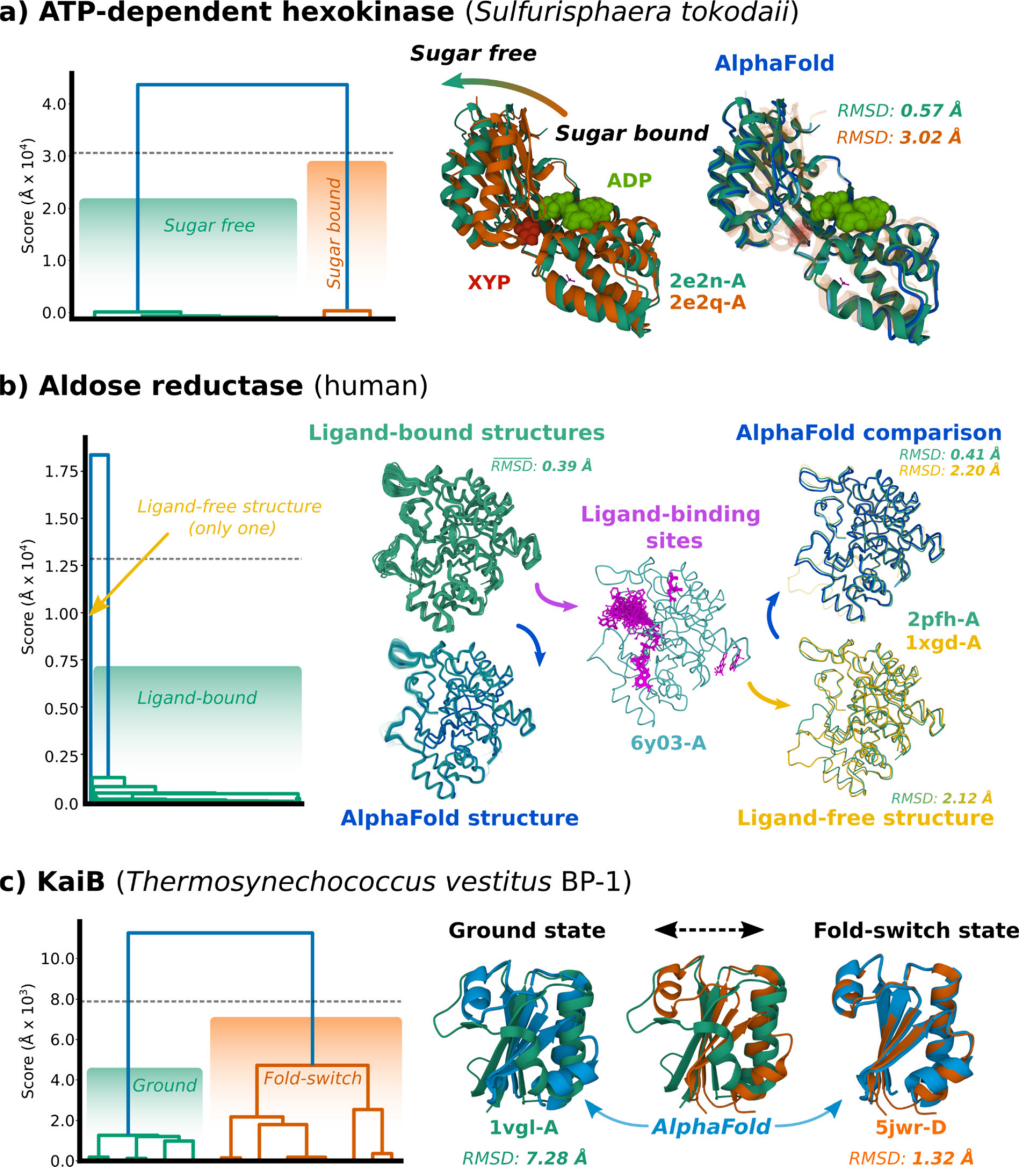
Notable examples of predicted conformational states by the PDBe-KB. (a) Clustering results in dendrogram (left) and structures (right) of the open–closed conformation change made by UniProt: Q96Y14. XYP (red) denotes β-d-xylopyranose and ADP (light green) denotes adenosine triphosphate, both bound to 2E2Q chain A. 2E2N chain A is an apo-form of the polypeptide. RMSD calculated between AlphaFold2 model and experimental structures. (b) Substrate promiscuity illustrated by consistent binding of diverse ligands (magenta), despite the polypeptide (UniProt: P15121) adopting a consistent conformation. Mean RMSD displayed for the collection of ligand-bound structures (top left), the AlphaFold2 structure to the two representative chains (top right), and between representative chains (bottom right). Structural variation between ligand-bound structures is relatively low, with a standard deviation in RMSD of 0.16 Å. Ligand-free structure (yellow) has a displaced loop in the Pro211-Asp230 region. (c) Fold-switch protein (UniProt: Q79V61) transitioning to control day–night cycle. Clustering dendrogram (left) with AlphaFold2 structure superposed alongside experimentally determined models. RMSD calculated between AlphaFold2 model and experimental structures.

The PDB provides a rich sampling of protein conformation space, where independently solved structures have identical sequences. Although a significant portion of the biologically meaningful conformation space has been captured, it is non-trivial to identify distinct conformations across all PDB entries.^80,81^ For example, hexokinase from *Sulfurisphaera tokodaii* (UniProt accession: Q96Y14) is the first glycolytic enzyme that initializes respiration and is essential during anaerobic conditions [Fig. 3(a)]. The kinase is moderately promiscuous to sugar substrates,^82^ allowing it to associate with glucose, mannose, glucosamine, xylose, and N-acetylglucosamine. Hexokinase adopts an open or a closed conformation, dependent on sugar binding, although ADP binding has a marginal effect on the protein's shape. Our automated pipeline can discern between the open and closed states, even identifying the open and closed chains solved within the asymmetric unit of 2E2Q [Fig. 3(a)].

Additionally, human aldose reductase (UniProt accession: P15121) accepts a diverse range of carbonyl-based substrates, reducing them to alcohol products using NADH as an electron source [Fig. 3(b)]. Many structures of this protein have been independently solved with a variety of ligands, providing information on the conformational heterogeneity within the holo state.^83^ Individual PDB entries fail to capture the structural heterogeneity in the β-sheet region spanning Val121-Arg156, but our pipeline can separate the only non-liganded structure in the PDB (1XGD) from all other ligand-bound chains. Superposition of all chains highlights a structural deviation in the unliganded structure within the Pro211-Asp230 loop.

Finally, the circadian rhythm protein KaiB (UniProt: Q79V61) helps regulate the day–night cycle in cyanobacteria and has been previously characterized as a fold-switch protein^79^ [Fig. 3(c)]. Associating with KaiA and KaiC, KaiB from *Thermosynechococcus vestitus* partakes in a concerted cycle of complex formation, autophosphorylation, and autodephosphorylation of KaiC, completing each oscillation every ∼24 h.^68^ KaiB adopts a homotetrameric ground state during the day and a thioredoxin-like “fold-switch” state at night. The fold-switch state is ordinarily stabilized upon oligomerization with KaiC and KaiB subunits, forming a multimeric complex.^68^ The clustering method described here identifies the structures solved in these two states and highlights that the protein's AlphaFold2 model from the AFDB is closer in conformation to the night-dominant fold-switch state.

## DISCUSSION

Exploring protein dynamics and conformational heterogeneity is essential for understanding molecular mechanisms and disease progression. However, capturing the full range of biologically relevant conformations—even for a single polypeptide—poses significant challenges beyond solving or predicting a static structure.^22,81^ Numerous experimental and computational methods characterize macromolecular dynamics, but a lack of standardization hinders comprehensive data integration. The next generation of integrative methodologies promises to combine diverse experimental data and computational techniques to achieve accurate and meaningful representations of conformational heterogeneity.^22^ Here, we have presented the method of clustering the static structures archived in the PDB. These clusters may depict some of the most stable, highly populated protein conformations (at 100% sequence identity) but cannot represent the complete free-energy topology nor the pathways traversed during conformational state transitions.

Nevertheless, even a high-accuracy representation of structural dynamics will be of limited value in answering biological questions unless contextualized with functional information. Attributing biological significance to conformational differences becomes much more challenging without annotations, such as ligand binding, oligomeric state, post-translational modifications, and point mutations, to name a few.^11–16^ When comparing more distantly related proteins, ontological annotations and domain mappings from resources such as CATH^84^ and SCOP^85^ can help systematically explore sequence–structure–function relationships. Automated annotation methods, utilizing structural motifs, domain composition, and comparative modeling, will be useful for predicting functions of uncharacterized proteins and their distinct conformations. Tools such as DALI,^86^ SSAP,^87^ and Foldseek^88^ are currently available for the identification of evolutionary relationships and functional similarities via structural comparison. As more conformations are determined experimentally—improving ensemble-model prediction algorithms—high-quality functional annotations necessitate integration to enable systematic analysis of structural diversity across different structure data archives. The PDBe-KB superposition and clustering pipeline presented here is a step toward this goal, but the collation of annotations is now needed before biological relevance can be systematically mapped to distinct protein conformations.

## CONCLUSION

Here, we present a deterministic data pipeline that clusters all proteins in the PDB archive based on model coordinates, independently of superposition. We demonstrated that the process can automatically identify distinct conformations, which, due to lack of standardized labeling in the archive, would otherwise be non-trivial to find in the PDB.

However, the lack of systematic, high-quality conformational state annotations impedes our understanding of the biological implications of protein dynamics. As such, functional annotations become available, and high-throughput mapping to conformations could be driven by initiatives such as the PDBe-KB consortium that has laid the groundwork for creating unified data access mechanisms and standard data exchange formats for a broad range of functional annotations.

Unlocking the potential of protein dynamics involves a multifaceted approach to understand their roles in biological mechanisms. It demands application of the innovative multimodal approaches seen by integrative modeling, combined with continued infrastructure improvement for high-throughput annotation and data access. As the field advances, these efforts will help with the development of novel therapeutic strategies and help us realize the relationship between protein sequence, structure, and function.

## Data Availability

The data that support the findings of this study are available within the article and its supplementary material.
